# Microalgae: emerging forces and prospects in the field of wound healing

**DOI:** 10.1093/burnst/tkag060

**Published:** 2026-09-02

**Authors:** Ruiyin Zeng, Haifeng Hu, Bobin Mi, Guohui Liu

**Affiliations:** Department of Orthopedics, Union Hospital, Tongji Medical College, Huazhong University of Science and Technology, 1277 Jiefang Avenue, Jianghan District, Wuhan 430022, China; Department of Orthopedics, Union Hospital, Tongji Medical College, Huazhong University of Science and Technology, 1277 Jiefang Avenue, Jianghan District, Wuhan 430022, China; Department of Orthopedics, Union Hospital, Tongji Medical College, Huazhong University of Science and Technology, 1277 Jiefang Avenue, Jianghan District, Wuhan 430022, China; Hubei Province Key Laboratory of Oral and Maxillofacial Development and Regeneration, Union Hospital, Tongji Medical College, Huazhong University of Science and Technology, 1277 Jiefang Avenue, Jianghan District, Wuhan 430022, China; Hubei Key Laboratory of Regenerative Medicine and Multi-disciplinary Translational Research, Union Hospital, Tongji Medical College, Huazhong University of Science and Technology, 1277 Jiefang Avenue, Jianghan District, Wuhan 430022, China; Department of Orthopedics, Union Hospital, Tongji Medical College, Huazhong University of Science and Technology, 1277 Jiefang Avenue, Jianghan District, Wuhan 430022, China; Hubei Province Key Laboratory of Oral and Maxillofacial Development and Regeneration, Union Hospital, Tongji Medical College, Huazhong University of Science and Technology, 1277 Jiefang Avenue, Jianghan District, Wuhan 430022, China

**Keywords:** Microalgae, Wound healing, Biomaterials, Photosynthetic oxygenation, Hydrogel

## Abstract

Viable microalgal cells have recently emerged as promising photosynthetic components of advanced wound healing systems because they can combine light-regulated oxygen generation, bioactive metabolite production, intrinsic imaging potential, and engineering flexibility within a single platform. In wounds with impaired or threatened healing such as burn wounds, traumatic full-thickness wounds, infected wounds, and chronic diabetic wounds, hypoxia, excessive inflammation, microbial burden, and impaired angiogenesis collectively hinder tissue repair and limit the efficacy of the currently employed therapies. In this review, the mechanistic basis and translational potential of microalgae in wound care is examined, with a focus on photosynthesis-driven oxygenation, antimicrobial and anti-inflammatory activities, antioxidant effects, immunomodulation, and bioimaging capability. We further summarize how viable microalgal cells or cell-derived products have been incorporated into hydrogels, 3D-bioprinted scaffolds, microneedles, electrospun matrices, coatings, and other biohybrid systems to achieve localized oxygen delivery, therapeutic molecule release, and multifunctional wound intervention. Importantly, we discuss the wound type–specific suitability of different microalgae-based platforms and critically compare them with existing oxygenation and antimicrobial strategies. We also highlight key barriers to clinical translation, including immune-mediated clearance, uncertain long-term survival and photosynthetic persistence in hostile wound microenvironments, limited light penetration, biosafety concerns, and challenges in manufacturing and standardization. Overall, microalgae-based wound therapeutics represent a highly promising but still early-stage strategy, and future progress will depend on mechanism-driven design, rigorous *in vivo* validation, and clinically relevant translational frameworks.

## Graphical Abstract

**Graphical Abstract:**
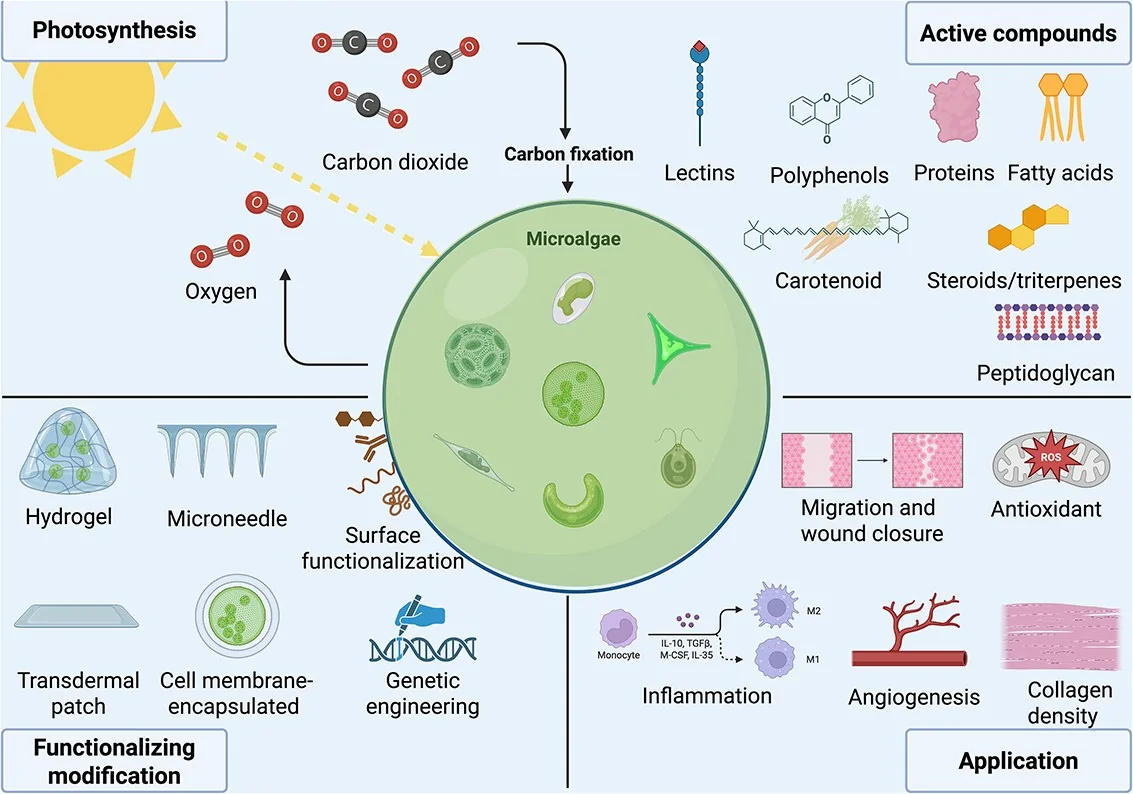

## Highlights

- Microalgae integrate renewable oxygen generation, bioactive molecules, and biomaterial engineering into multifunctional wound therapeutics.
- Living microalgae and processed microalgal products exhibit distinct mechanisms, advantages, and translational considerations.
- Wound phenotype–specific design is critical for optimizing the therapeutic performance of microalgae-based interventions.
- Microalgae are best positioned as multifunctional adjunctive platforms alongside established wound therapies rather than as stand-alone replacements.
- Clinical translation depends on resolving challenges related to cell persistence, immune interactions, light availability, biosafety, manufacturing, and standardization.

## Background

Cutaneous wound healing requires coordinated hemostasis, inflammation, proliferation, and remodeling. Failure of this sequence can lead to chronic inflammation, infection, fibrosis, graft loss, and the development of nonhealing ulcers [1, 2]. Biological obstacles differ among wound types. Burn wounds combine tissue necrosis, barrier loss, inflammatory amplification, and infection risk; traumatic and full-thickness wounds may require rapid hemostasis and structural reconstruction; and diabetic wounds are characterized by neuropathy, vascular insufficiency, hypoxia, oxidative stress, and impaired immune function [3, 4]. The currently employed treatment strategy involves debridement, infection control, moisture-balancing dressings, pressure offloading, revascularization, grafting, and adjunctive oxygen therapy [5, 6]. These measures remain indispensable, yet difficult wounds often contain several mutually reinforcing abnormalities. Hypoxia impairs fibroblast and keratinocyte activity, collagen maturation, and angiogenesis; infection increases oxygen consumption and protease activity; and prolonged oxidative and inflammatory signaling further damages the microvasculature [2, 7]. Consequently, a single treatment method is unlikely to me*et al*l clinical needs. These limitations have driven growing interest in intelligent biomaterials that are not only structurally supportive but also biologically active, adaptive, and capable of modulating the wound microenvironment in a more dynamic and multifunctional way [8].

Within this context, microalgae have attracted increasing attention as photosynthetically active components that can be used as biomaterials that advance wound healing. In this review, the term “microalgae” is used pragmatically to include eukaryotic microalgae and oxygenic cyanobacteria while recognizing their taxonomic, cellular, and biosafety differences. “Viable microalgal cells” refer specifically to metabolically active eukaryotic microalgae or oxygenic cyanobacteria intentionally incorporated into a therapeutic construct. Viable microalgal cells can convert light into oxygen and biomass, secrete vesicles or recombinant proteins, and respond dynamically to environmental cues. Nonliving extracts contain pigments, polysaccharides, fatty acids, proteins, and peptides with potential antioxidant, antimicrobial, or regenerative activity [9, 10]. This duality enables two fundamentally different product classes: viable photosynthetic constructs that require control of cell dose and illumination and processed microalgal products that behave more like conventional biologics or biomaterial additives.

Moreover, enthusiasm for microalgae-based wound therapeutics should be balanced by careful and critical evaluation. Their actual *in vivo* performance may be strongly influenced by wound type and microenvironmental stressors, including low pH, protease-rich exudates, oxidative stress, immune cell infiltration, limited light penetration, and mechanical instability at the wound bed [3, 4, 11, 12]. Likewise, the relative contributions of oxygen generation, algal metabolites, biomaterial carriers, and combination therapeutics have not been clearly separated in previous studies. These unresolved questions are central to assessing whether microalgae can offer meaningful advantages over existing wound oxygenation or antimicrobial strategies in clinically relevant settings.

Several reviews have summarized microalgal biomedicine or photosynthesis-based oxygenation [9, 10, 13]. The present review differs by focusing specifically on wound repair; the effects mediated by viable microalgal cells are distinguished from those mediated by cell-free microalgal products, evidence is organized according to wound phenotype, and microalgal systems with established oxygen and antimicrobial strategies are critically compared. Particular attention is given to the following questions concerning clinical translation: whether cells remain functional in exudative and inflamed wounds, how they are cleared, how antimicrobial claims should be interpreted, and which combinations offer advantages beyond increasingly sophisticated cell-free or conventional dressings.

## Review

### Why microalgae are uniquely relevant to wound repair

The term “microalgae” is frequently used pragmatically, but eukaryotic microalgae and oxygenic cyanobacteria differ in their cellular organization, cell wall composition, genetic systems, and potential inflammatory contaminants [14, 15]. In biomedical applications, commonly studied species include Chlorella, *Chlamydomonas reinhardtii*, Spirulina/Arthrospira, *Haematococcus pluvialis*, Porphyridium, and diatoms. Their relevance to wound repair arises from a combination of biological functions that are rarely present in a single therapeutic platform: oxygen generation, the production of bioactive metabolites, optical activity, surface modifiability, and compatibility with biomaterial engineering strategies.

#### A modular therapeutic platform

The most important feature of microalgae is not any single molecule but their modularity. A construct containing viable microalgal cells may simultaneously produce dissolved oxygen, consume carbon dioxide, have intrinsic fluorescence properties, and serve as a chassis for recombinant protein secretion. A processed product can instead exploit phycocyanin, carotenoids, polysaccharides, fatty acids, peptides, or extracellular vesicles without the persistence of viable microalgal cells [16–19]. Material engineering adds a third layer by controlling retention, mechanical protection, hydration, and mass transfer and release. In addition, the seed-lot provenance, medium composition, illumination spectrum, photoperiod, temperature, carbon supply, growth phase, and harvesting method can alter cell size, pigment content, membrane composition, and metabolic output [14–16, 20, 21].

#### Species and product selection

*Chlamydomonas reinhardtii* is widely used because it is genetically tractable, motile, and compatible with several hydrogel systems. Chlorella species are robust oxygen producers and have been incorporated into patches, microneedles, and symbiotic hydrogels. *Synechococcus elongatus* and related cyanobacteria are attractive for oxygen production and extracellular vesicle secretion, but their prokaryotic nature changes the host response and containment considerations. *Haematococcus pluvialis* provides a switchable phenotype: green cells support photosynthesis, whereas stress-induced red cells accumulate astaxanthin. Arthrospira (Spirulina), Euglena, and Phaeodactylum have been investigated mainly as biomass or extraction sources [22–26]. Diatoms, with their silica-based frustules and optical properties, have potential relevance in biosensing and imaging [27].

#### Viable microalgal cells versus processed products

Systems containing viable photosynthetic microalgae can renew oxygen production during illumination but require maintenance of microalgal cell viability, nutrient and gas exchange, a suitable temperature, and protection from immune and antimicrobial stress. Processed systems are easier to sterilize, standardize, and store but cannot generate oxygen and may lose activity during extraction. Cell-free extracellular vesicles occupy an intermediate category: they retain biological cargo and may promote angiogenesis without the requirements of whole organisms [19, 28].

Microalgal metabolites span diverse classes. Sulfated and neutral polysaccharides can affect hydration, cell adhesion, and inflammation; carotenoids and phenolic fractions can combat oxidative stress; fatty acids and peptides may have antimicrobial or promigratory effects; and polydeoxyribonucleotides may support skin regeneration [18, 23,29–44]. Much of this evidence originates outside of cutaneous wound models.

Extracellular vesicles are a promising cell-free category. Vesicles from *S. elongatus* accelerated experimental cutaneous repair and angiogenesis, and nanoalgosome studies revealed that microalgae release vesicle-like particles that can be isolated and taken up by mammalian cells [16, 45]. More recently, Haematococcus-derived vesicles enriched in astaxanthin and proteins associated with antioxidant defense were incorporated into an adhesive hydrogel for diabetic wound and peripheral nerve repair [46]. These systems may avoid the light and containment requirements associated with viable whole-cell microalgal products, but vesicle identity, coisolated material, cargo potency, dose normalization, and scalable purification remain unresolved [47]. The product-defining functions, advantages, limitations, and most plausible wound-care uses of the major microalgal therapeutic classes are summarized in Table 1.

**Table 1:** Design logic for major classes of microalgae-based wound therapeutics

**Product class**	**Principal function**	**Main advantages**	**Main limitations**	**Refs.**
Viable photosynthetic microalgal cells in hydrogels or scaffolds	Light-controlled dissolved oxygen; optional secretion of therapeutic drugs	Renewable local function; spatial retention; programmable output	Light dependence; viability; containment; variable oxygen dose	[48, 52, 69, 76]
Processed biomass or extracts	Antioxidant, anti-inflammatory, antimicrobial, or trophic activity	Easier sterilization and storage; no viable-organism release	Batch variability; uncertain active dose; no sustained oxygen	[9, 23, 51, 90]
Purified pigments, polysaccharides, lipids or peptides	Defined bioactivity and material functionalization	Better analytical control; compatible with conventional manufacturing	Isolation cost; degradation; activity may be context dependent	[18, 32, 54, 79]
Microalgal extracellular vesicles	Paracrine delivery of proteins, lipids, and nucleic acids	Cell-free; nanoscale delivery; potential angiogenic activity	Cargo heterogeneity; isolation and potency assays not standardized	[19, 28, 46]
Genetically engineered cells	Oxygen plus local production of growth factors or other proteins	Multiproduct “living factory”; potentially sustained local delivery	Gene stability, dose control, ecological containment, regulatory complexity	[60–62]

### Mechanistic basis of microalgae-mediated wound healing

Microalgae-based interventions act through partially overlapping oxygenation, antimicrobial, redox, immune, and regenerative pathways (Figure 1). These pathways should be interpreted according to product class and experimental controls because viable microalgal cells, cell-derived compounds, light, carrier materials, and coloaded therapeutics may contribute independently or synergistically.

**Figure 1:**
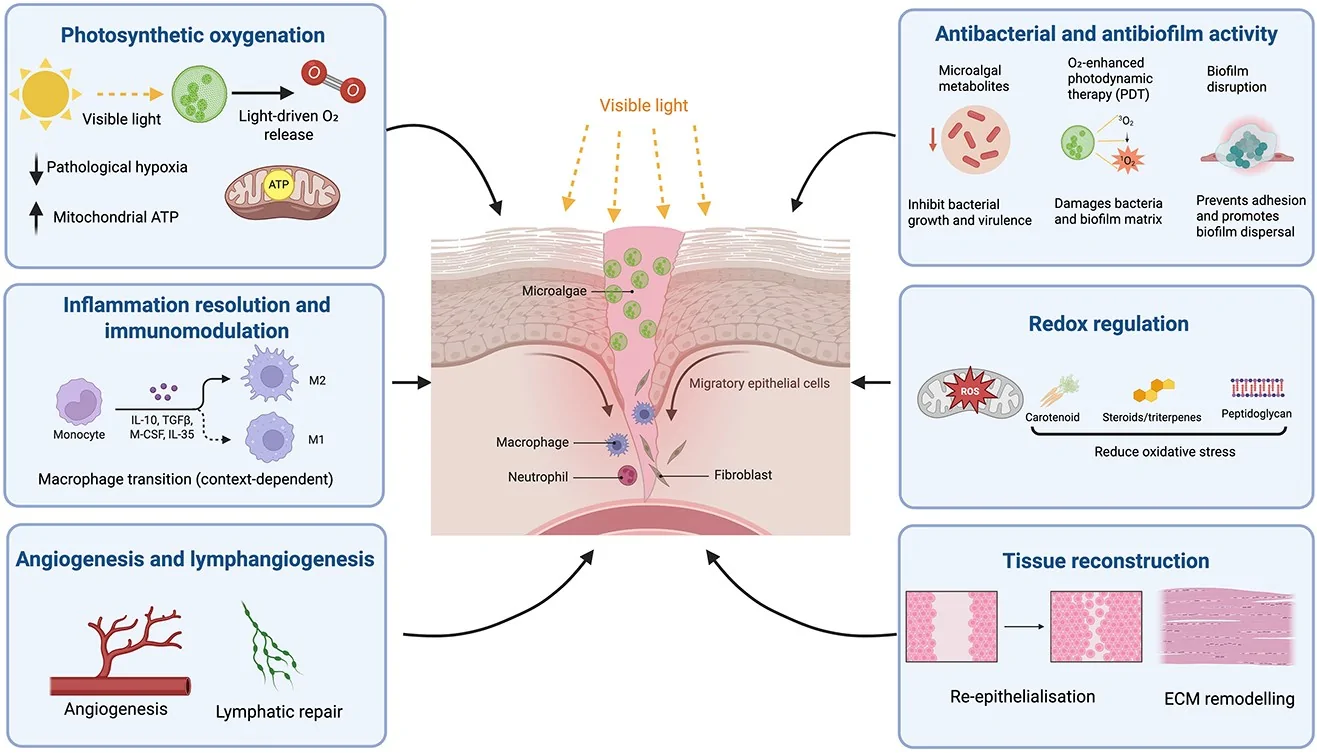
Mechanisms of microalgae-mediated wound repair. Microalgae-based interventions influence wound healing through multiple interconnected pathways. Photosynthetic oxygenation alleviates tissue hypoxia and supports cellular metabolism, angiogenesis, and collagen synthesis. Bioactive metabolites contribute to antioxidant, anti-inflammatory, and antimicrobial activities. These biological effects collectively regulate immune responses, reduce oxidative stress, promote tissue reconstruction, and improve the homeostasis of the wound microenvironment. The relative contribution of each pathway depends on the specific microalgal species, product format, biomaterial carrier, and therapeutic design. *ATP* adenosine triphosphate, *ECM* extracellular matrix, *IL* interleukin, *M-CSF* macrophage colony-stimulating factor, *PDT* photodynamic therapy, *ROS* reactive oxygen species, *TGF-β* transforming growth factor beta

#### Photosynthetic oxygenation

Hypoxia is a central barrier in burn wounds, traumatic tissue defects, infected wounds, and diabetic ulcers. Oxygen supports mitochondrial adenosine triphosphate production, oxidative killing by neutrophils, epithelial migration, fibroblast proliferation, collagen hydroxylation, and angiogenesis. Chronic wounds become hypoxic because vascular disruption and edema reduce delivery, whereas activated immune cells and microorganisms increase consumption [2, 7]. Microalgae address this imbalance through light-driven water oxidation, releasing molecular oxygen directly into the surrounding aqueous phase. In a diabetic mouse model, a hydrogel patch containing viable *S. elongatus* cells delivered dissolved oxygen through the skin more efficiently than topical gaseous oxygen did and promoted angiogenesis, wound closure, and graft survival [48].

Oxygen generation is nevertheless conditional rather than intrinsically continuous. The output depends on the wavelength, irradiance, exposure schedule, photosynthetic state, cell density, carbon availability, temperature, matrix diffusion, and local balance between production and consumption. Excessive light can cause heating or photoinhibition, whereas excessive oxygen can increase oxidative injury. Programmed Haematococcus gels illustrate how illumination can be used intentionally: higher irradiance supports photothermal antibacterial action, whereas lower irradiance favors photosynthetic oxygenation [24].

#### Antimicrobial and antibiofilm effects

The antibacterial effects of microalgae-based systems are regulated by several distinct mechanisms. Microalgal peptides, free fatty acids, pigments, and phenolic metabolites may disrupt microbial membranes, interfere with metabolism, or alter redox homeostasis [22, 49, 50]. Gelatin fibers loaded with microalgal extracts and spirulina-based coatings have been verified to effectively inhibit the growth of common wound pathogens *in vitro* [26, 51]. However, the generation of oxygen by viable photosynthetic microalgae alone is not a reliable antibacterial treatment. Restoring oxygen generation may improve host defense and potentiate oxygen-dependent photodynamic therapy, but it does not replace debridement or targeted antimicrobial therapy. Many high-performing constructs therefore include ciprofloxacin, silver, copper sulfide, probiotics, or photosensitizers [52–55]. In 2025, a Chlorella-based oxygenator coupled photosynthetic oxygen production with a porphyrin photosensitizer to improve the efficacy of antibiofilm photodynamic therapy in diabetic wounds, exemplifying a mechanistically coherent combination [56]. Related photosynthetic-bacteria hydrogels also accelerate repair in infected-wound models [57]. These findings support the general principle that biological oxygen production can be combined with local infection control.

In conclusion, this multimodal antibacterial potential is attractive, but it also creates interpretive complexity. In many published systems, antibacterial activity cannot be attributed to microalgae alone. The observed effect may result from the combined action of oxygenation, photodynamic reactions, the material matrix, and additional antimicrobial agents. Microalgae-based systems may therefore be best understood as multifunctional adjunctive platforms rather than replacements for established antimicrobial therapy.

#### Regulation of oxidative stress and inflammation

Reactive oxygen species (ROS) are required for signaling and antimicrobial defense, but sustained ROS accumulation damages membranes, proteins, and DNA and prolongs inflammation. Carotenoids such as astaxanthin, beta-carotene, and fucoxanthin can quench radicals and influence inflammatory pathways; phycocyanin and selected polysaccharides also show antioxidant and anti-inflammatory activity [18, 29, 32, 58]. These effects are particularly relevant to diabetic wounds, where oxidative stress is prominent.

In chronic wounds, sustained proinflammatory macrophage activation is associated with high protease activity, excessive ROS production, and impaired tissue reconstruction. Microalgae-containing materials can also modify macrophage responses. Euglena-based hydrogels promote repair without excessive inflammation, and programmed Haematococcus gels reduce inflammatory signaling while supporting reparative macrophage phenotypes [24, 25]. Composite systems frequently have strong amounts of ROS-scavenging components. For example, a dual-crosslinked silk gel combining viable microalgal cells with cerium oxide nanoparticles was constructed in 2025, linking photosynthetic oxygenation to ROS clearance and M2-associated immunoregulation in diabetic wounds [59].

#### Angiogenesis, cell migration, and matrix remodeling

Improved oxygenation can stabilize cellular energy metabolism and reduce maladaptive hypoxic signaling, thereby supporting endothelial tube formation, fibroblast migration, and collagen deposition. Microalgae also provide oxygen-independent signals. Extracellular vesicles secreted by *S. elongatus* accelerate cutaneous repair by promoting angiogenesis [28]. Microalgal proteins, lipids, and polysaccharides have increased keratinocyte or fibroblast migration in experimental models, whereas the use of Parachlorella-derived fatty acids promoted excisional and burn healing *in vivo* [23, 44]. These findings suggest that processed products may be preferable when trophic signaling rather than oxygenation is the primary goal. Genetic engineering extends this concept. *Chlamydomonas reinhardtii* has been engineered to produce human VEGF, PDGF-B, and SDF-1, enabling simultaneous oxygen and growth factor delivery from sutures or hydrogels [60, 61]. Lymphatic repair is an underdeveloped mechanism. Impaired lymphatic drainage can promote edema and inflammatory cell retention. Transgenic *Synechococcus* sp. PCC 7002 engineered to produce hyaluronic acid supported lymphatic endothelial cell survival and tube formation in hypoxic dermal regeneration scaffolds [62]. This expands the mechanisms of photosynthesis-related gene therapy beyond restoring oxygen generation and blood vessel angiogenesis.

#### Intrinsic fluorescence properties and wound monitoring

The direct application of microalgal fluorescence to wound imaging remains in its early stages. Therefore, this topic should be framed as an emerging opportunity rather than an established wound care function. Chlorophyll and accessory pigments generate autofluorescence, allowing microalgal location and viability to be monitored without an added label. The fluorescence intensity can also change with temperature, photosynthetic stress, and pigment degradation [63, 64]. In wound systems, the most realistic near-term role is quality control: confirming cell distribution, retention, and loss of photosynthetic activity in transparent or superficial constructs. *Diatom biosilica* can enhance fluorescence-based detection, but previous studies have focused mainly on immunoassays rather than wound monitoring [27]. Microalgal fluorescence should also be distinguished from clinical bacterial fluorescence. Currently employed imaging devices detect host or bacterial fluorophores, not therapeutic algae, under defined excitation wavelengths. A 2026 randomized trial reported that real-time fluorescence-guided debridement had a lower residual bacterial area and shorter healing time than conventional debridement [65]. This provides a benchmark for actionable optical information but does not validate algal fluorescence as a bacterial sensor. Future work should determine whether endogenous fluorescence can reliably indicate changes in microalgal viability, oxygen-generating activity, or the wound microenvironment *in vivo* [64, 66, 67].

### Biomaterial and device platforms

Microalgae are rarely applied to wounds as free cells. Instead, they are usually incorporated into biomaterials or device-like platforms that improve retention, protect biological function, and enable localized therapeutic action. These platforms include hydrogels, bioprinted microalgae-containing scaffolds, microneedles, electrospun fibers, coatings, encapsulation systems, genetically engineered constructs, and biohybrid microrobots. Each platform has distinct advantages and limitations, and their suitability depends on the wound type, depth, exudate level, illumination feasibility, and clinical workflow (Figure 2).

**Figure 2:**
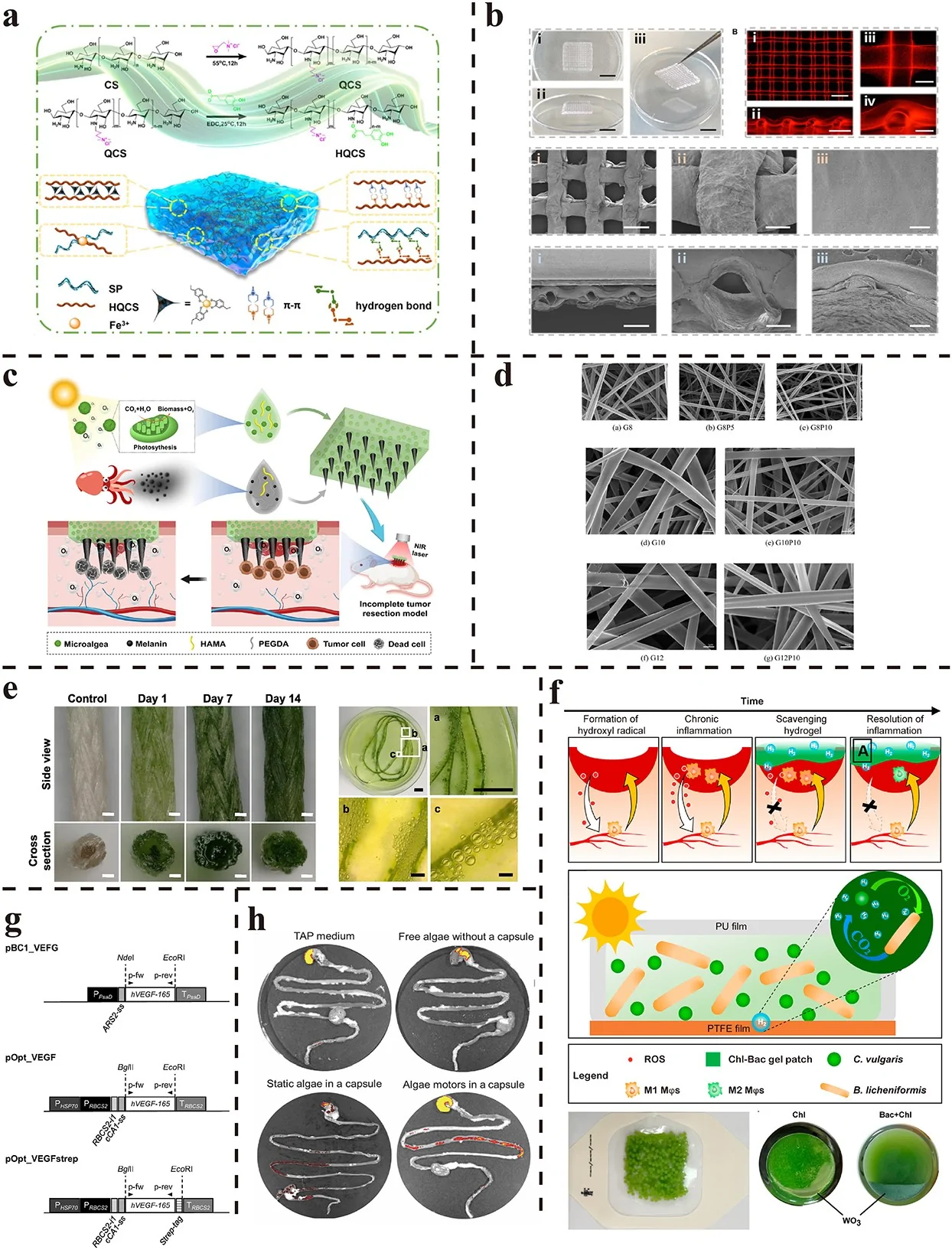
Representative biomaterial and bioengineering platforms for microalgae-based wound therapy and related delivery applications. (**a**) Synthesis of hydrocaffeic-acid-grafted quaternized chitosan and formation of the HQCS-SP hydrogel through ferric-ion coordination, π–π interactions and hydrogen bonding (Copyright 2024, MDPI) [68]. *CS* chitosan, *HQCS* hydrocaffeic-acid-grafted quaternized chitosan, *QCS* quaternized chitosan, *SP* spirulina-derived protein. (**b**) Macroscopic, fluorescence, and scanning electron microscopy characterization of *P. notoginseng* saponin–containing hollow fibrous scaffolds fabricated by microfluidic bioprinting (Copyright 2023, AAAS) [75]. (**c**) This section depicts the creation of photosynthetic microneedle patches designed for localized oxygen delivery and postoperative melanoma therapy (Copyright 2024, Springer Nature) [78]. *HAMA* methacrylated hyaluronic acid, *NIR* near-infrared, *PEGDA* poly(ethylene glycol) diacrylate. (**d**) Scanning electron microscopy images of electrospun gelatin nanofibres prepared with or without *Phaeodactylum tricornutum* extract. (Copyright 2014, Elsevier) [26]. G8, G10, and G12, gelatin solutions at 8%, 10%, and 12% (w/v), respectively; P5 and P10, *P. tricornutum* extract at 0.5% and 1.0% (w/v), respectively. (**e**) Surface and cross-sectional images showing the growth of *C. reinhardtii* within polyglactin sutures over 14 days, together with macroscopic and microscopic evidence of microalgal retention and oxygen-bubble formation (Copyright 2018, Elsevier) [60]. (**f**) Design and proposed mechanism of a symbiotic *Chlorella vulgaris*–*Bacillus licheniformis* gel patch (Copyright 2022, American Chemical Society) [86]. *Bac + Chl* coculture of *B. licheniformis* and *C. vulgaris*, *Chl C. vulgaris* alone, *Chl-Bac* symbiotic *Chlorella–Bacillus* construct, *Mφs* macrophages, *PTFE* polytetrafluoroethylene, *ROS* reactive oxygen species. (**g**) Expression constructs used for the production and secretion of human vascular endothelial growth factor isoform 165 by genetically engineered *C. reinhardtii* (Copyright 2019, Springer Nature) [61]. *hVEGF* human vascular endothelial growth factor isoform 165. (**h**) Representative *ex vivo* fluorescence images of the gastrointestinal tract 5 h after oral administration of Tris-acetate-phosphate medium, free algae, static algae in a capsule or motile algae in a capsule. This panel illustrates a related microalgal delivery strategy outside wound care (Copyright 2022, AAAS) [89]. *TAP* Tris-acetate-phosphate

#### Hydrogels and patches

Hydrogels are the dominant platform because they retain cells, maintain hydration, and permit the diffusion of light, gases, and metabolites. Alginate, GelMA, chitosan, hyaluronic acid, silk, and synthetic networks are commonly used [25, 52, 68, 69]. The matrix design should meet the following competing requirements: high optical transmission and gas diffusion, sufficient mechanical integrity, adhesion to an irregular wound, exudate management, and nontraumatic removal. Rapid, mild crosslinking is particularly important because heat, extreme pH, and reactive initiators reduce cell viability. For instance, a photocrosslinked carboxymethyl-chitosan gel formed within 30 s at room temperature and combined *C. reinhardtii* with ciprofloxacin, improving oxygenation, increasing the expression of angiogenic markers and promoting the closure of diabetic wounds [52]. The amoeba-inspired polyvinyl alcohol (PVA)/carrageenan system established in 2026 combined the self-healing, and motion-sensing properties of Chlorella and ampicillin and had increased injectability [70].

Several pathological axes have been increasingly addressed in the construction of advanced hydrogels. ROS-responsive *C. reinhardtii* gels have been shown to deliver basic fibroblast growth factor [71], while pH-modulating Synechococcus hydrogels have been shown to promote oxygenation and puerarin release [72]. In 2025, a bilayer was constructed to spatially separate photosynthetic cells from a photothermal silver–fulvic acid antibacterial layer, an approach that may protect cell function while reducing the biofilm burden [73]. Such architectures are rational when the components require different optical or chemical environments.

#### 3D printed and scaffold-based systems

3D bioprinting enables patient-specific geometry, spatial control of cell density and formation of channels that improve mass transfer. *In situ*–printed hollow-fiber scaffolds containing microalgae relieved hypoxia and accelerated full-thickness wound repair in diabetic mice [74]. Microfluidic printing has also been used to combine microalgae with *Panax notoginseng* saponins [75]. For translation, the relevant advantages should include conformity to complex defects, predictable oxygen distribution, surgical handling, and compatibility with grafting.

Dermal regeneration templates seeded with *C. reinhardtii* have strong clinical promotion potential. These scaffolds use an established structural matrix while adding a temporary photosynthetic function. This design is especially relevant to deep full-thickness defects and burn-scar reconstruction, where the construct can support granulation before autografting [76, 77].

#### Microneedles and minimally invasive devices

Microneedles bypass the superficial diffusion barrier and can inject cells, oxygen, or drugs into the skin at a defined depth. Chlorella-loaded antibacterial microneedles improved oxygenation and healing in infected diabetic wounds [53]. Their strengths include reproducible geometry and local microchannels, but their weaknesses, including needle fracture, insertion pain, microbial translocation, and uneven contact on irregular wounds, require evaluation. Postsurgical melanoma systems demonstrate the technical versatility of this type of microneedle system [78].

#### Fibers, coatings, microspheres, and cell-derived formulations

Electrospun fibers can mimic aspects of extracellular matrix architecture and provide a large surface area for cell interaction, drug loading, and wound coverage. Microalgal peptide/poly-ε-caprolactone (PCL)–carrageenan fibers and Phaeodactylum extract–loaded gelatin mats showed antibacterial and wound-relevant activity [26, 79]. Coating strategies may also be used to deposit microalgae-derived materials onto wound-contacting surfaces. For example, plasma processing has converted Spirulina biomass into ultrathin bioactive coatings [51]. Microspheres can localize purified phycocyanin or biomass and permit injectable or composite delivery [54, 80]. These nonliving formats may offer a shorter regulatory path because sterilization and storage resemble conventional dressings, although active-component identity and release kinetics still require definition.

Encapsulation and lipid-carrier approaches can protect microalgae or microalgae-derived compounds from harsh wound environments while enabling controlled release or localized activity. Liposomes, nanostructured carriers, and polymer capsules can improve stability, prolong retention, and modulate release kinetics. Encapsulation of live microalgae may also reduce direct immune recognition while preserving oxygen and metabolite exchange [81, 82].

#### Genetically engineered viable microalgal systems

Genetic engineering expands the therapeutic potential of microalgae by enabling the expression of growth factors, antimicrobial peptides, cytokine-modulating molecules, or environmental-response systems. *Chlamydomonas reinhardtii* is particularly relevant because it is a well-established model organism for genetic manipulation. The chloroplast and nuclear genomes of *C. reinhardtii* can be engineered to express recombinant proteins [61, 83]. Photosynthetic sutures have been used to deliver oxygen together with VEGF, PDGF-B, or SDF-1 [60]. Future constructs could use inducible promoters, secretion tags, and nutrient dependence to tune local output.

However, therapeutics containing genetically engineered viable microalgae introduce substantial biosafety and regulatory challenges. Key concerns include genetic stability, uncontrolled persistence, horizontal gene transfer, environmental release, and patient-specific immune responses. For wound healing, engineered microalgae should therefore be developed with containment strategies, defined functional lifetimes, and rigorous safety evaluations [62, 84].

#### Symbiotic materials containing multiple viable microorganisms

Microalgae cocultured with other microorganisms offer innovative solutions for wound healing. These symbiotic biomaterials can integrate the advantages of different microbes to exert synergistic effects, providing more effective solutions for chronic wound healing. Chlorella and *Bacillus subtilis* were combined in a hydrogel to supply oxygen and suppress pathogens [55]. Lichen-inspired printed constructs are similar to paired microalgae with probiotics [85]. There are also symbiotic alga–bacteria constructs that produce hydrogen to accelerate diabetic wound healing [86].

These systems may sustain reciprocal metabolism, but they substantially increase complexity. Species ratios can drift; antimicrobial activity may harm therapeutic algae; and two viable microorganisms require independent identity, purity, potency, and containment specifications. They should therefore be advanced only when the symbiosis provides a measurable benefit that cannot be achieved by a cell-free antimicrobial treatment.

#### Targeted delivery, sensing, and engineering lessons from other organs

Microalgal motility, magnetic modification, surface conjugation, and intrinsic fluorescence have enabled the development of biohybrid microrobots and targeted delivery in tumor, lung, and gastrointestinal models [66,87–89]. These studies demonstrate directional transport, payload attachment, imaging, and barrier traversal, but they are not evidence of wound healing. Open wounds rarely require long-range microswimming, and guided particles may be less practical than a conformal removable dressing. The wound-relevant opportunity is local spatial control. Microalgae could be patterned within a scaffold to match oxygen demand, restricted to a retrievable layer, or combined with deformation sensors that indicate dressing integrity. Adaptive Chlorella hydrogels involve the integration of oxygenation, antibacterial treatment, and motion monitoring [70].

### Wound-specific applications

Wound phenotypes associated with impaired healing are biologically heterogeneous, and the therapeutic value of microalgae-based systems is therefore unlikely to be uniform across all wound types. Rather than broadly stating that microalgae “promote wound healing,” it is more clinically meaningful to consider which microalgae-based platforms are best suited to specific wound microenvironments. Burn wounds, traumatic full-thickness defects, infected wounds, and chronic diabetic wounds differ substantially in terms of oxygen demand, exudate burden, inflammatory intensity, microbial contamination, vascular status, and tissue accessibility. These differences shape both the potential advantages and the practical limitations of interventions involving viable microalgal cells.

#### Burn wounds

Burns require stage-specific management. Early deep burns contain necrotic tissue and a high risk of microbial invasion; definitive treatment may require excision, grafting, and reconstruction. Photosynthetic oxygenation is unlikely to replace these interventions but may support prepared wound beds, dermal substitutes, and graft survival. Parachlorella-derived fatty acids improved experimental excisional and burn wounds, whereas a fish-collagen/gelatin emulgel containing Chlorella extract and silver increased neovascularization and re-epithelialization in a rat full-thickness burn model [23, 90].

Superficial partial-thickness burns may heal through surviving adnexal structures, whereas deep burns often require excision, temporary coverage, and grafting. Eschar, blood, and topical antimicrobial agents can attenuate light or reduce algal viability. Large burns also involve systemic fluid, temperature, and inflammatory problems that cannot be addressed by a local photosynthetic dressing [2, 3]. Extract creams and lipid-carrier formulations provide topical concepts, although *in vitro* migration and antioxidant assays should not be equated with burn efficacy [82, 91].

In short, for acute burns, nonliving extracts may be easier to deploy than viable constructs because topical antimicrobials, exudates, and frequent dressing changes can compromise cell survival. Scaffolds containing viable microalgal cells may be more appropriate after excision when the wound is clean, when vascular reconstruction is needed, and when controlled illumination is feasible.

#### Traumatic and full-thickness wounds

Acute traumatic wounds prioritize hemostasis, debridement, closure, and restoration of tissue architecture. Microalgae-based hydrogels may add oxygenation and trophic support after contamination has been controlled. Euglena/chitosan–hyaluronic acid hydrogels and compound Spirulina hydrogels accelerated full-thickness repair in rodents [25, 68]. 3D-printed scaffolds containing viable microalgal cells are attractive for treating irregular defects, especially when a temporary matrix is needed before grafting [74].

Human photosynthetic scaffold studies are most relevant to this category. Eight patients with clean full-thickness wounds received *C. reinhardtii*–seeded dermal regeneration scaffolds, followed by grafting. No deleterious local or systemic immune response was detected for 90 days [76]. Another case involved treating a 56-year-old patient with elbow scar contracture by first excising the scar tissue before covering the wound with a photosynthetic scaffold exposed to light to promote local oxygen production; subsequently, an autologous graft was implanted. After 17 months of follow-up, the photosynthetic scaffold exhibited long-term safety and applicability in dermis regeneration [77]. These observations demonstrate the feasibility of this technique in selected patients, but its efficacy cannot be isolated from the use of a dermal template, surgery, or autografting.

#### Infected wounds

Infected wounds require source control and pathogen-directed therapy. Microalgae can serve as oxygenators, carriers, or sources of antimicrobial metabolites, but few systems have been compared with standard antimicrobial dressings. Chlorella microneedles, ciprofloxacin-loaded photosynthetic gels, phycocyanin–copper sulfide microspheres, and Spirulina coatings have been shown to reduce bacterial burden in preclinical models [51–54]. Oxygen-producing systems may be especially useful when paired with photodynamic therapy because local oxygen depletion limits ROS generation [56]. The critical translational question is whether treatment eradicates biofilms without causing photothermal or oxidative tissue injury. In addition, the use of constructs containing viable therapeutic bacterial cells needs additional caution in immunocompromised or extensively burned patients.

#### Diabetic chronic wounds

Diabetic wounds are the most intensively studied indication because hypoxia, oxidative stress, persistent inflammation, infection, and impaired angiogenesis align with the proposed functions of microalgae. The seminal dissolved-oxygen patch improved healing and graft survival in diabetic mice [48]. Subsequent systems include programmed photothermal therapy, ROS-responsive growth factor delivery, pH modulation, microneedle penetration, and symbiotic infection control [24, 53, 55, 71, 72]. The microalgae–silk/CeO2 gel and bilayer microalgae–silver hydrogel constructed in 2025 further illustrate the shift toward multiaxis microenvironmental regulation [59, 73]. Other platforms include microalgae-powered microrobots, metformin/microalgae chitosan sponges, algae–bacteria hydrogen-producing dressings, oxygen-assisted antibiofilm photodynamic therapy (PDT), adaptive Chlorella hydrogels, and microalgal-vesicle hydrogels [46, 70, 86, 88, 92]. However, most evidence comes from small, splinted, or unsplinted rodent excisional wounds. These models do not reproduce neuropathy, repetitive pressure, arterial disease, callus, or polymicrobial biofilms of diabetic foot ulcers. Before clinical trials, microalgal dressings should be tested with offloading and debridement in large-animal or human *ex vivo* models and compared with advanced standard care.

#### Postsurgical wounds and composite defects

Photosynthetic sutures and scaffolds can localize oxygen and growth factors along incision lines or beneath grafts [60]. This is conceptually attractive for compromised closures, irradiated tissue, and reconstructive surgery. Nevertheless, oxygen requirements vary spatially, and a closed incision receives less light than an open wound does. Removable or externally illuminated constructs are therefore more realistic than deeply implanted free cells. Each wound phenotype is linked to its dominant biological barrier, the most plausible microalgal strategy, the required standard-care cointervention, and the principal evidence gap in Table 2. 

**Table 2:** Wound-specific suitability of representative microalgae-based strategies

**Wound phenotype**	**Dominant barrier**	**Preferred microalgal strategy**	**Required cointervention**	**Key evidence gap**	**Refs.**
Excised deep burn or graft bed	Hypoxia, tissue loss, graft failure	Photosynthetic dermal scaffold or oxygenating hydrogel	Excision, infection control, grafting	Graft take and scar outcomes in comparative burn studies	[76, 77, 90]
Superficial burn	Oxidative stress, inflammation, barrier loss	Standardized pigment, lipid or extract dressing	Moisture and infection management	Dose, sensitization and superiority to modern dressings	[23, 82, 91]
Clean traumatic/full-thickness defect	Structural loss and transient hypoxia	Bioprinted scaffold containing viable microalgal cells or a conformal hydrogel	Debridement and closure or reconstruction	Large-animal handling and functional outcomes	[25, 68, 74]
Infected wound	Biofilm and high oxygen consumption	Oxygenator plus a defined antimicrobial or PDT	Debridement and culture-directed therapy	Polymicrobial biofilm eradication and recurrence	[52–54, 56]
Diabetic chronic wound	Hypoxia, ROS, inflammation, angiogenic failure	Multifunctional hydrogel or microneedle containing viable microalgal cells	Offloading, vascular assessment, glucose and infection control	Clinically representative models and head-to-head trials	[24, 48, 59, 60,71–73]
Postsurgical compromised closure	Local ischemia and dehiscence risk	Photosynthetic suture or removable patch	Standard closure and perfusion optimization	Light delivery beneath closed tissue and device retrieval	[60, 78]

*PDT* photodynamic therapy, *ROS* reactive oxygen species

### Comparison with current oxygenation and antimicrobial strategies

Hyperbaric oxygen therapy increases systemic plasma oxygen levels under pressure and may benefit selected hypoxic diabetic wounds, but access, cost, treatment burden, and oxygen-related adverse effects limit its broad use [45, 93]. Microalgae-based oxygenation may offer a more localized and potentially sustained alternative for accessible wounds. However, the evidence base for microalgae remains far less mature than that for established oxygen therapies. Their function also depends on light exposure and biological viability. Therefore, microalgae should not currently be presented as a replacement for hyperbaric oxygen therapy but as a potential local oxygenation strategy for selected wound contexts.

Topical oxygen therapy delivers oxygen locally, and there is randomized evidence in diabetic foot and chronic wounds [94, 95]. The key difference is that topical oxygen therapy is device-driven, whereas microalgal oxygenation is biologically generated. This not only creates opportunities for sustained local production but also introduces variability. If microalgae lose viability, lack sufficient illumination, or are cleared by the host, oxygen generation decreases. Direct comparative studies are needed to determine whether the duration, depth, or biological effects of microalgal oxygenation are superior to those of topical oxygen therapy.

Chemical oxygen-generating dressings and oxygen carriers, such as hemoglobin-based or perfluorocarbon-based systems, avoid cell viability and light dependence but may have finite payloads, burst release, or reactive byproducts [96–98]. Nonmicroalgal oxygen-generating hydrogels are becoming more sophisticated and can be combined with electrical stimulation or catalytic materials [99]. Microalgae differ in their ability to produce dissolved oxygen repeatedly during illumination and by supporting additional biological functions. This is their most distinctive advantage over oxygen-carrying dressings. However, this advantage only exists if photosynthetic activity persists *in vivo*. Therefore, the central comparison should not be “oxygen carrier versus oxygen generator” in principle but “finite reservoir versus viable and photosynthetically functional microalgal generator” under real wound conditions.

Antimicrobial dressings containing silver, iodine, or antiseptic polymers have established manufacturing pathways and broad activity [100–102]. Their main advantage is direct antimicrobial activity. Their limitation is that they may not correct hypoxia, impaired angiogenesis, oxidative stress, or chronic inflammation. Microalgae-based platforms may offer broader microenvironmental regulation by combining antimicrobial action with oxygenation and bioactive molecule delivery. However, current evidence does not support the replacement of standard antimicrobial dressings with microalgae-based systems in infected wounds. Instead, microalgae may be most useful as part of combination platforms, where oxygenation, photodynamic effects, algal metabolites, and conventional antimicrobial agents work together. Future studies should include clinically relevant bacterial models, biofilm assays, and comparator groups using standard antimicrobial dressings. The delivery mechanism, antimicrobial contribution, principal strength, and main limitations of microalgal products are compared with those of established adjunctive wound strategies in Table 3. 

**Table 3:** Comparison of microalgae-based systems with established adjunctive wound strategies

**Strategy**	**Oxygen delivery**	**Antimicrobial contribution**	**Main strength**	**Main limitation**	**Refs.**
HBOT	Systemic, intermittent, high partial pressure	Indirect support of host defense	Clinical infrastructure and defined dosing	Cost, access, systemic exposure and repeated sessions	[45, 93]
Topical oxygen	Local gaseous oxygen, continuous or cyclical	Limited direct effect	Portable and clinically available	Variable penetration and wound-sealing requirements	[94, 95]
Chemical oxygen-generating dressing	Local finite oxygen release	Depends on additives	No viable microbial cells or external illumination	Burst kinetics and potentially reactive byproducts	[99]
Oxygen-carrier dressing	Stores or transports oxygen	Depends on additives	Can be formulated as a conventional dressing	Finite capacity and material-specific safety issues	[13]
Live microalgal hydrogel or scaffold	Local dissolved oxygen during illumination	Indirect unless algae or additives are antimicrobial	Renewable, controllable, and multifunctional	Light and viability dependence; containment and manufacturing	[48, 56, 69]
Microalgal extract dressing	None	Compound-dependent direct activity	Simpler storage and sterilization; antioxidant cobenefit	Batch variability and uncertain active constituent	[23, 51, 79]

*HBOT* hyperbaric oxygen therapy

### Translational barriers and safety considerations

Despite the growing promise of microalgae-based wound therapeutics, translation from experimental systems to clinically usable products remains at an early stage. Recent preclinical studies demonstrate important proof-of-concept capabilities, but several key barriers continue to limit confidence in their real-world applicability.

#### Immune-mediated clearance and host response

Unlike inert biomaterials, viable microalgal cells are xenogeneic biological entities and are therefore likely to interact dynamically with innate immune cells, inflammatory mediators, wound exudates, and local tissue defense mechanisms. Phagocytic uptake, neutrophil-rich inflammatory responses, complement activation, and oxidative injury may all shorten the therapeutic lifetime of microalgae at the wound site. Even when overt toxicity is not observed, progressive immune-mediated attrition may substantially reduce oxygen production and other intended functions.

A phase I scaffold study revealed no harmful local or systemic immune response, including no increase in major circulating T-cell subsets, and biopsies revealed tissue integration [76]. The treated scaffolds lost their initial green color before grafting, suggesting a decrease in pigment or cell viability, but the study did not establish a quantitative clearance curve. Future work should track viable cells—not fluorescence alone—using culture, species-specific nucleic acid assays, and histology at defined intervals. Studies should distinguish immune tolerance from the absence of exposure and should include immunocompromised, infected, and burn-relevant settings.

#### Survival in hostile wound microenvironments

Wound environments differ markedly from standard microalgal culture conditions. Temperature fluctuations, acidic or alkaline pH, osmotic stress, protease-rich exudate, topical antimicrobial agents, nutrient limitation, high levels of ROS, bacterial competition, and mechanical shear can reduce microalgal viability and photosynthetic output. Occlusive dressings may restrict carbon dioxide exchange, whereas blood, eschar, and exudate attenuate incident light. Consequently, the viability and oxygen production measured in culture medium or buffer may overestimate the functional persistence of viable microalgal cells in wounds.

Current solutions focus on protecting viable microalgal cells while preserving light, gas, nutrient, and metabolite exchange. Hydrogel encapsulation and semipermeable membrane patches can retain microalgal cells, buffer mechanical stress, maintain hydration, and permit the bidirectional transport of water and dissolved gases [48, 69, 81]. Porous, hollow, or 3D-printed architectures can increase the surface-to-volume ratio, shorten diffusion distances, and increase the exposure of embedded microalgae to light and carbon sources [74]. Adaptive and self-healing matrices may reduce structural damage caused by compression and shear at mobile wound sites [70]. Microneedles can position the oxygen-producing compartment or its dissolved-oxygen output closer to hypoxic tissue, whereas symbiotic constructs may provide reciprocal metabolic or antimicrobial support [53, 55]. However, symbiotic systems also increase manufacturing, potency, and containment complexity. For clinical translation, these material strategies should be combined with strain selection, controlled microalgal cell density, defined carbon and nutrient supplementation, retrievable or replaceable dressings, and a prespecified functional treatment window.

#### Light penetration and phototoxicity

Visible light is absorbed and scattered by blood, melanin, eschar, exudate, and tissue; thus, conventional photosynthetic constructs are most suitable for superficial or surgically exposed wounds. Simply increasing irradiance is unlikely to provide a satisfactory solution because excessive light can cause local heating, photoinhibition, and ROS-mediated tissue injury. Currently employed strategies therefore prioritize device geometry, optical transmission, and controlled illumination rather than irradiance alone. Thin and transparent hydrogels or semipermeable patches permit external illumination while allowing the dissolved oxygen generated by viable microalgal cells to diffuse toward the wound [48, 69]. Porous, hollow, or high-surface-area printed scaffolds shorten the optical path length and position viable microalgal cells closer to the illuminated surface [74]. Microneedles can reduce the distance over which dissolved oxygen must diffuse and can deliver an oxygen-producing compartment closer to superficial hypoxic tissue, although they do not eliminate photon attenuation in deeper tissue [53, 78]. Wavelength-matched and intermittent illumination, combined with temperature monitoring, may further reduce photothermal injury and photoinhibition. 

**Table 4:** Translational evidence and next decisive study

**Evidence level**	**Representative finding**	**What it establishes**	**What remains unresolved**	**Refs.**
*In vitro* or *ex vivo*	Oxygen production, cell compatibility, and antibacterial or antioxidant activity	Mechanistic plausibility	Therapeutic dose and performance in real wound fluid	[28, 69, 80]
Rodent wounds	Improved closure, angiogenesis, and collagen deposition in diabetic or infected wounds	Preclinical efficacy signal	Human relevance, recurrence, and standard-care comparison	[24, 48, 53, 59]
Healthy-volunteer skin	Photosynthetic hydrogel tolerated without irritation	Short-term cutaneous compatibility	Efficacy and safety in open or infected wounds	[69]
Phase I full-thickness wounds	Eight patients showed feasibility without harmful immune response	Initial safety of implanted photosynthetic scaffolds	Comparative efficacy, clearance kinetics, and scalable manufacture	[76]
Long-term clinical case	Scaffold integration, followed by grafting and functional reconstruction	Feasibility of staged reconstruction in one selected patient	Generalizability and contribution of the microalgal component	[77]
Periodontal randomized trial	Spirulina peptide gel improved healing-related outcomes	Human signal for a processed product	Generalizability to cutaneous burns or chronic wounds	[106]

Additional approaches borrowed from optogenetics and photosynthetic engineering include red-shifted excitation, near-infrared-responsive biological switches, and molecular antennas that broaden the usable light spectrum [103, 104]. These approaches remain exploratory for wound oxygenation because their conversion efficiency, depth-dependent oxygen output, photothermal effects, material safety, and manufacturing complexity have not been adequately established.

#### Biosafety of viable and genetically engineered strains

Although preliminary evidence suggests that some microalgae-based constructs may be locally tolerated, biosafety assessments remain insufficiently mature [69, 77]. Potential risks include contamination, opportunistic infection, toxin or endotoxin-like inflammatory activity, horizontal gene transfer, environmental release, and the persistence of engineered traits. Risks differ markedly between eukaryotic algae and cyanobacteria and must be assessed strain by strain. Genetic constructs should avoid clinically important antibiotic resistance markers and incorporate auxotrophy, kill switches, or dependence on synthetic nutrients. Safety testing should include identity, purity, microbial contaminants, toxin screening, local irritation, sensitization, systemic dissemination, shedding, reproductive or environmental persistence when relevant, and interactions with antibiotics. For engineered products, transgene stability and output must be measured throughout the intended treatment period and after stress.

#### Manufacturing, storage, sterilization, and quality control

Materials containing viable microalgal cells cannot generally be terminally sterilized by many conventional methods without loss of function. Manufacturing may therefore require aseptic processing, closed cultivation, and release testing for bacteria, fungi, mycoplasma, and adventitious agents. The critical quality attributes include strain identity, viable-cell dose, photosynthetic efficiency, oxygen output, matrix mechanics, optical transmission, therapeutic-protein expression, and the absence of harmful metabolites.

For combination products, antibiotic potency, nanoparticle state, and sensor performance must remain stable together with the cells. Stability protocols should measure function rather than appearance or total cell count. Processed products have a simpler path but still require source control, contaminant limits, chemical fingerprints, and mechanism-linked potency assays [81, 105]. In addition, evidence that the effects of microalgal vesicle preparations are attributable to vesicles rather than soluble pigments or proteins is needed [16, 46].

Storage is another bottleneck. Fresh constructs offer high viability but short shelf life and complex logistics; dried or cryopreserved formulations may reduce function after rehydration. Nonliving extracts have simpler logistics but need chemical fingerprints and potency assays to control batch variation.

### Clinical evidence and future directions

Clinical evidence remains preliminary. The first-in-human phase I study included eight patients with clean full-thickness wounds and demonstrated the feasibility and 90-day safety of a *C. reinhardtii*–seeded dermal regeneration scaffold [76]. This long-term case report supports the compatibility between autografting and functional reconstruction [77]. A photosynthetic alginate hydrogel was also tested on the intact skin of 20 healthy volunteers without irritation, supporting material handling, and short-term cutaneous compatibility rather than wound-healing efficacy [69]. In 2025, a split-mouth randomized trial of a Spirulina-derived peptide gel after periodontal flap surgery reported improved clinical healing and inflammatory outcomes, providing human evidence for a processed microalgal product in a specialized surgical wound [106].

These observations answer questions regarding feasibility but not those regarding comparative efficacy. Scaffold recipients also received a dermal template, surgery, and autografting; the healthy-volunteer study did not involve open wounds, and the periodontal wounds differed from burns and diabetic ulcers. No adequately powered randomized trial has established that a dressing containing viable microalgal cells improves durable cutaneous closure over the same carrier and standard care. Recent syntheses similarly identify manufacturing, dosing, and clinical validation as major gaps [60, 105].

These studies do not yet justify the broad clinical translation of these products. The next generation of trials should compare a defined microalgal product with an identical carrier and with the current advanced care treatment. Three types of therapeutics appear to be the most likely to be developed. First, retrievable photosynthetic scaffolds can be developed for clean, exposed full-thickness defects can build on existing human feasibility. Second, cell-free microalgal extracts, pigments, or vesicles can be developed as standardized adjuncts for burns or chronic wounds. Third, oxygen-producing systems can be developed and combined with therapies whose efficacy is demonstrably oxygen limited, such as PDT, provided that the combined benefit exceeds that of either component. The current translational evidence hierarchy and the next decisive study required to establish added clinical value are summarized in Table 4.

## Conclusions

Microalgae offer an unusual combination of renewable oxygen production, bioactive metabolites, genetic programmability, and compatibility with advanced biomaterials. Their value in wound repair is most convincing when the selected function matches a defined wound barrier: photosynthetic oxygenation to alleviate tissue hypoxia, standardized extracts to combat oxidative or inflammatory injury, and oxygen-assisted antimicrobial therapy to heal infected wounds. The literature supports the feasibility of microalgae-based treatments to target and heal wounds, whereas superiority over the standard care treatments remains unproven. Translation will require quantitative control of the viable-cell dose, oxygen flux, illumination, persistence, containment, manufacturing, and retrieval, together with matched-carrier and standard-care comparators. With these safeguards, retrievable photosynthetic constructs and standardized cell-free microalgal products may become focused adjuncts rather than replacements for established wound care.

## Data Availability

Data sharing is not applicable for this article, as no datasets were generated or analyzed during the current study.

## Abbreviations

AMP, antimicrobial peptide; bFGF, basic fibroblast growth factor; EV, extracellular vesicle; HBOT, hyperbaric oxygen therapy; HIF-1α, hypoxia-inducible factor-1 alpha; NIR, near-infrared; PDGF-B, platelet-derived growth factor subunit B; PDT, photodynamic therapy; ROS, reactive oxygen species; SDF-1, stromal cell-derived factor-1; TOWT, topical wound oxygen therapy; VEGF, vascular endothelial growth factor.
